# Identification of cytochrome P450 isoenzymes involved in the metabolism of 23-hydroxybetulinic acid in human liver microsomes

**DOI:** 10.1080/13880209.2019.1701500

**Published:** 2019-12-23

**Authors:** Ying Zhou, Jinhua Wen, Guangji Wang

**Affiliations:** a Department of Pharmacy, The First Affiliated Hospital of Nanchang University, Nanchang, China; b Key Laboratory of Drug Metabolism and Pharmacokinetics, China Pharmaceutical University, Nanjing, China

**Keywords:** Medicinal herb, metabolic kinetics, enzyme inhibition, herb–drug interaction

## Abstract

**Context:**

23-Hydroxybetulinic acid (23-HBA), a major active constituent of *Pulsatilla chinensis* (Bunge) Regel (Ranunculaceae), exhibits potential antitumor activity. Its metabolism, however, has not yet been studied.

**Objective:**

This study focuses on the metabolism of 23-HBA *in vitro* by human liver microsomes.

**Materials and methods:**

The metabolic kinetics of 23-HBA (0.5–100 µM) and the effects of selective CYP450 (CYP1A2, CYP2C9, CYP2C19, CYP2D6, and CYP3A4) inhibitors on metabolism of 23-HBA were evaluated in human liver microsomes incubation system and then determined by LC-MS method. The Michaelis–Menten parameters *K_m_* and *V_max_* were initially estimated by analysing Lineweaver–Burk plot. The clearance (*CL_int_*) was also calculated.

**Results:**

The *V_max_, K_m_,* and *CL_int_* of 23-HBA were 256.41 ± 11.20 pmol/min/mg, 11.10 ± 1.07 μM, and 23.10 ± 1.32 μL/min/mg, respectively. The metabolism of 23-HBA was significantly inhibited by furafylline (0.05 μM, *p* < 0.01) and ketoconazole (0.02 μM, *p* < 0.05). Ticlopidine (1.3 μM, *p* < 0.05) could inhibit the metabolism of 23-HBA, while the other inhibitors (sulfaphenazole and quinidine) showed nonsignificant inhibition on the metabolism of 23-HBA.

**Discussion and conclusions:**

This is the first investigation of the metabolism of 23-HBA in human liver microsomes. The *in vitro* study indicates that CYP1A2 and CYP3A4 are mainly involved in the metabolism of 23-HBA. Special attention should be given to the pharmacokinetic and clinical outcomes when 23-HBA was co-administrated with other compounds mainly undergoing CYP1A2/CYP3A4-mediated metabolism. Further studies are needed to evaluate the significance of this interaction and strengthen the understanding of traditional Chinese medicine.

## Introduction

*Pulsatilla chinensis* (Bunge) Regel (Ranunculaceae) is a botanical with a long history of medical use in China, the roots are widely used in the traditional Chinese medicine for adjunctive treatment of malaria, vaginal trichomoniasis, intestinal amoebiasis, bacterial infections, and malignant tumour (Cheng et al. 2008).

**Figure 1.**
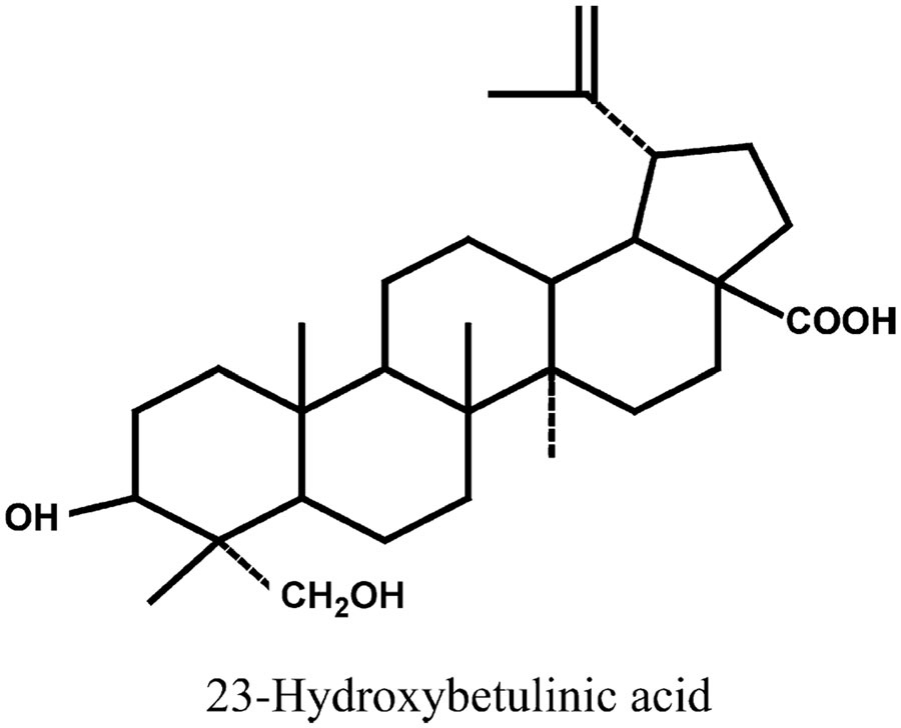
Chemical structure of 23-hydroxybetulinic acid (23-HBA).

23-Hydroxybetulinic acid (23-HBA, Figure 1), an isolated pentacyclic triterpene, is the major active constituent of *Pulsatilla chinensis* (Ye et al. 1996). 23-HBA has been found to have cytotoxicity against a variety of tumour cell lines (Ji et al. 2002; Zhou et al. 2007; Zhang et al. 2015), such as the human non-small-cell lung cancer cell line NCI-H460, human gastric carcinoma cell line SGC7901 and human hepatocellular carcinoma cell line HepG2 and possesses synergistic effects on the cytotoxicity of doxorubicin *in vitro* and *in vivo* (Zheng et al. 2010). Herein, 23-HBA has a high potential to be developed as a novel safe chemosensitizer. However, despite the pharmacological importance of 23-HBA, there are no reports about its metabolism pathways, and especially whether it is metabolized by CYP450 enzyme and which subtypes are involved.

This study investigates the metabolic kinetics of 23-HBA and confirm which subtypes of CYP450 are responsible for the metabolism of 23-HBA in human liver microsomes.

## Materials and methods

### Chemicals and reagents

23-HBA (99% purity) was a kind gift from Professor WC Ye (Jinan University, Guangzhou, China). Furafylline (Fur), sulfaphenazole (Sul), ticlopidine (Tic), quinidine (Qui), ketoconazole (Ket), and NADPH were purchased from Sigma-Aldrich (St. Louis, MO, USA). *In vitro* CYP H-class 10-Donor pooled human liver microsomes (Lot No. X008061) were obtained from Research Institute for Liver Disease Co., Ltd. (Shanghai, China).

### Incubation system

The incubation mixture, with a total volume of 200 μL, contained 100 mM potassium phosphate buffer (pH 7.4), NADPH generating system (1 mM NADP^+^, 10 mM glucose 6-phosphate, 1 unit/mL glucose-6-phosphate dehydrogenase and 5 mM MgCl_2_), human liver microsomes (0.2 mg/mL), CYP isoform-specific inhibitors and 23-HBA (1 μM). Incubation time, substrate concentration and liver microsomal concentration were all optimized in the study. The reaction was initiated by adding the NADPH-generating system after a 5 min pre-incubation at 37 °C. After incubation for 15 min in a shaking water bath, the reaction was terminated by the addition of ethyl acetate (800 μL, containing 5 μM oleanolic acid as internal standard). The mixture was kept on ice until it was centrifuged at 20,000 *g* for 10 min at 4 °C. Aliquots of supernatants were transferred for LC-MS analysis. Control incubations without NADPH or without substrate or without microsomes were included to ensure that metabolism of 23-HBA were microsomes and NADPH dependent. All incubations were performed in triplicate.

### LC-MS method

LC-MS was carried out on a Shimadazu (Japan) LCMSD quadrupole mass spectrometer equipped with a series 2010 HPLC system. A Zorbax Extend-C_18_ column (50 mm × 2.1 mm, 5 µm, Agilent, USA) was used to separate 23-HBA. The mobile phase, consisted of acetonitrile (A) and water containing 0.05% (v/v) triethylamine (B) (A:B = 70:30, v:v), was run at a flow rate of 0.2 mL/min. Negative ion electrospray ionization (ESI) was used to form deprotonated molecules at m/z 471.20 of 23-hydroxybetulinic acid and *m*/*z* 455.35 of the internal standard oleanolic acid. Selected ion monitoring (SIM) was used. The optimum ESI conditions for 23-hydroxybetulinic acid and oleanolic acid included a probe voltage of 4500 V, a detector gain of 1600 V, a nitrogen nebulizer pressure of 35 psi, and nitrogen drying gas temperature of 250 °C at 4.5 L/min.

### Kinetic study

To evaluate kinetic parameters, 23-HBA (0.5, 1, 2, 5, 10, 20, 50, 100 μM) was incubated with pooled human liver microsomes (0.2 mg/mL) for 15 min. Preliminary experiments were performed to make sure that the disappearance of 23-HBA was in the linear range of both reaction time and the concentration of microsomes. The apparent *V_max_* and *K_m_* values were calculated from non-linear regression analysis of experimental data according to the Michaelis–Menten equation using GraphPad Prism software (version 5.0, GraphPad Software, Inc., San Diego, CA). Accordingly, *CL_int_* of 23-HBA was calculated from the value of *V_max_/K_m_*.

### Chemical inhibition study

Chemical inhibition studies were carried out by adding different human CYP inhibitors to the incubation mixture of 23-HBA (1 µM) before the addition of the NADPH-generating system. The inhibitors utilized were as follows: Fur (CYP1A2, 0.05, 0.1, 0.2, 0.5, 1, 2 μM), Sul (CYP2C9, 0.01, 0.02, 0.05, 0.1, 0.2, 0.5 μM), Tic (CYP2C19, 0.08, 0.16, 0.32, 0.65, 1.3, 2.6 μM), Qui (CYP2D6, 0.01, 0.02, 0.05, 0.1, 0.2, 0.4 μM) and Ket (CYP3A4, 0.01, 0.02, 0.05, 0.1, 0.2, 0.4 μM). The concentrations of inhibitors used in the study were verified to inhibit the specific activities of corresponding CYP isoforms in human liver microsomes (Weaver et al. 2003; Lim et al. 2013).

## Results

### The enzyme kinetics of 23-HBA

**Figure 2.**
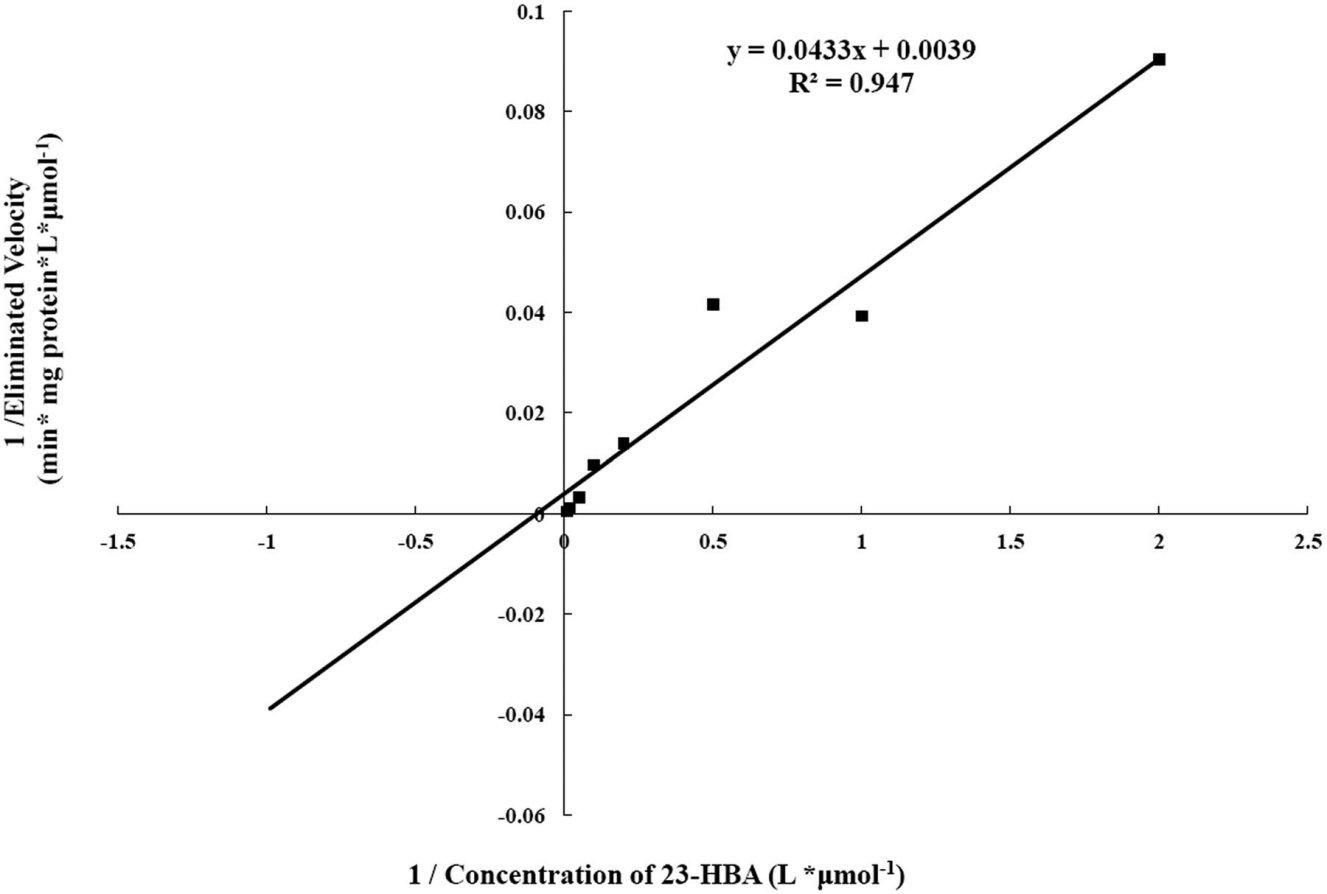
Lineweaver–Burk plot of kinetic analysis of the elimination of 23-HBA in the human liver microsomes. All data represent the mean of the incubations (performed in triplicate).

Preliminary studies indicated that the elimination of 23-HBA was linear up to 15-min incubation time when the concentration of liver microsomes was 0.2 mg/mL at 37 °C. Thus, the kinetic study of 23-HBA in liver microsomes was evaluated using a protein concentration of 0.2 mg/mL and an incubation time of 15 min. Under the experimental conditions used, the metabolism of 23-HBA in human liver microsomes obeyed typical Michaelis–Menten kinetics (shown in Figure 2). The kinetic parameters (apparent *V_max_* and *K_m_*) were calculated to be 256.41 ± 11.20 pmol/min/mg and 11.10 ± 1.07 μM. Accordingly, apparent *CL_int_* was 23.10 ± 1.32 μL/min/mg.

### The effects of inhibitors on the metabolism of 23-HBA

**Figure 3.**
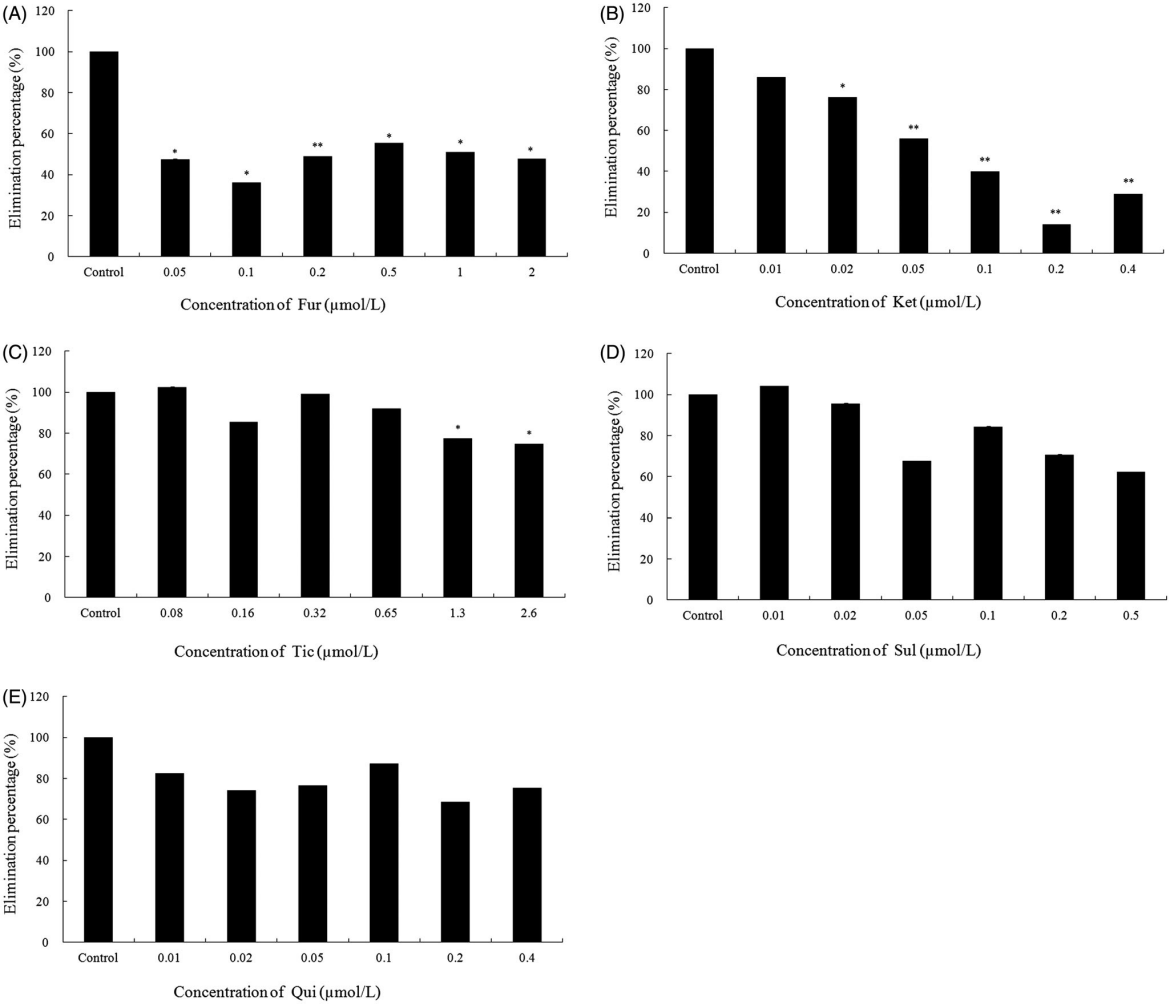
Effects of various CYP450s selective inhibitors on metabolism of 23-HBA (1 µM) in human liver microsomes: (A) furafylline (Fur); (B) ketoconazole (Ket); (C) ticlopidine (Tic); (D) sulfaphenazole (Sul); (E) quinidine (Qui). Significance indicated by: *p < 0.05, **p < 0.01 versus control. Data are presented as mean ± SD of three independent experiments.

The effect of various chemical inhibitors on the metabolism of 23-HBA was investigated in pooled human liver microsomes. Among the selective inhibitors of five CYP isoforms, furafylline (the selective inhibitor of CYP1A2) could significantly inhibit the disappearance of 23-HBA at all the tested concentration (Figure 3(A)). Also, ketoconazole (the selective inhibitor of CYP3A4) could significantly inhibit the metabolism of 23-HBA at low concentration (0.02 μM, *p* < 0.05) (Figure 3(B)). Ticlopidine (the selective inhibitor of CYP2C19) produced a slight inhibitory effect on the metabolism of 23-HBA at high concentration (>1.3 μM, *p* < 0.05) (Figure 3(C)). However, sulfaphenazole (the selective inhibitor of CYP2C9) (Figure 3(D)) and quinidine (the selective inhibitor of CYP2D6) (Figure 3(E)) showed non-significant inhibition on the metabolism of 23-HBA through the tested concentration. It showed that CYP1A2 and CYP3A4 were mainly involved in the metabolism of 23-HBA. CYP2C19 contributed to the metabolism of 23-HBA to some extent, while CYP2C9 and CYP2D6 might have no effect on the metabolism of 23-HBA.

## Discussion

Herbal medicines are widely used in the treatment of different diseases because of the efficacy and low toxicity. *Pulsatilla chinensis* has been used as adjuvant in chemotherapy in traditional Chinese medicine for a long time. 23-HBA is the major active constituent of *Pulsatilla chinensis* and has a high potential to be developed as a novel safe chemosensitizer. Studies on the metabolism of a new drug have an important role for determining the safety and therapeutic potential of the drug (Lin and Lu 1997; Fang et al. 2011). Moreover, drug metabolism is the major route of drug clearance and CYP450 is the main factor most frequently responsible for inter-individual differences in pharmacokinetics owing to polymorphic or inducible expression. Therefore, it is necessary to identify the drug-metabolizing enzymes involved in metabolism of 23-HBA.

Cytochrome P450s is the most important drug-metabolizing enzyme family, among which CYP3A, CYP2C and CYP2D are responsible for metabolism of about 50%, 25%, and 20% of drugs (Rendic and Carlo 1997; Guengerich 2006). To understand about the metabolism of 23-HBA *in vitro* by human liver microsomes, we firstly investigated the characteristics of enzyme kinetic of 23-HBA. The Michaelis–Menten parameters *K_m_* and *V_max_* were initially estimated by analysing Lineweaver–Burk plot. The clearance (*CL_int_*) was also calculated.

Chemical inhibition study showed that CYP1A2 and CYP3A4 were mainly involved in the metabolism of 23-HBA. CYP2C19 contributed to the metabolism of 23-HBA to some extent, while CYP2C9 and CYP2D6 might have no effect on the metabolism of 23-HBA. 23-HBA possesses synergistic effects on the cytotoxicity of doxorubicin *in vitro* and *in vivo* (Zheng et al. 2010). As we known, doxorubicin is a typical substrate of CYP3A4, the synergism may be associated with the inhibition of the metabolism of doxorubicin by 23-HBA.

Therefore, special attention should be paid on pharmacokinetic and clinical outcomes when 23-HBA was co-administrated with other compounds mainly undergoing CYP1A2/CYP3A4-mediated metabolism.

## Conclusions

The present study is the first to investigate the metabolism of 23-HBA in human liver microsomes. It was found that CYP450s appeared to be involved in the metabolism of 23-HBA. CYP1A2 and CYP3A4 were the major drug-metabolizing CYP enzymes involved in the metabolism of 23-HBA. The results are helpful for a deeper understanding of metabolic and pharmacokinetic behaviour of 23-HBA. In the later, further study can be made to explore the effect of 23-HBA on the activity of CYP450s *in vitro* and *in vivo*, so as to provide some useful information for safe and effective use of 23-HBA in clinical practice and strengthen the understanding of traditional Chinese medicine.
